# Donor-specific photoisomerization in visible squaraine dyes regulates the fluorescence quantum yield over two orders of magnitude

**DOI:** 10.1080/14686996.2026.2732506

**Published:** 2026-10-12

**Authors:** Michael Bauer, Elodie Didier, João Pedro Ferreira Assunção, Lola Richeboeuf, Frank Nüesch, Daniel Rentsch, Daniele Padula, Roland Hany

**Affiliations:** a Empa, Swiss Federal Laboratories for Materials Science and Technology, Laboratory for Functional Polymers, Dübendorf, Switzerland; b Institute of Chemistry and Chemical Engineering, Ecole Polytechnique Fédérale de Lausanne, EPFL, Lausanne, Switzerland; c Institute of Materials Science and Engineering, Ecole Polytechnique Fédérale de Lausanne, EPFL, Lausanne, Switzerland; d Dipartimento di Biotecnologie, Chimica e Farmacia, Universitià di Siena, Siena, Italy

**Keywords:** Squaraine dye, polymethine dye, essential states model, photoisomerization

## Abstract

Isomerization reactions in photoexcited polymethine dyes represent a familiar non-radiative deactivation channel that competes with light emission. Here, absorption and emission spectra are reported for a comprehensive series of synthesized 1,2- and 1,3-substituted, methine-bridged squaraines utilizing indoline-, benzothiazole- and quinoline-derived donor heterocycles. Absorption mirror emission spectra and Stokes shifts are small and largely solvent-independent, implying minor geometric differences between the ground and excited state from which emission occurs. Indoline- and benzothiazole-based 1,2-squaraines show fluorescence quantum yields of ≈5%, increasing to ≈40% for 1,3-squaraines. However, emission is almost completely quenched for any compound that incorporates the strong quinoline donor. Efficient non-radiative deactivation is supported by quantum chemical calculations along the twisting motion of the lateral quinoline group in the polymethine bridge. Design rules are derived from calculations using a simple three-state model, and facile photoisomerization is predicted for asymmetric squaraines with very different donors, as well as for symmetric dyes with strong donors.

## IMPACT STATEMENT

Fast photoisomerization quenches the fluorescence in symmetric donor–acceptor–donor squaraines with strong donors, and in asymmetric squaraines when the donors are very different.

## GRAPHICAL ABSTRACT

**Figure:**
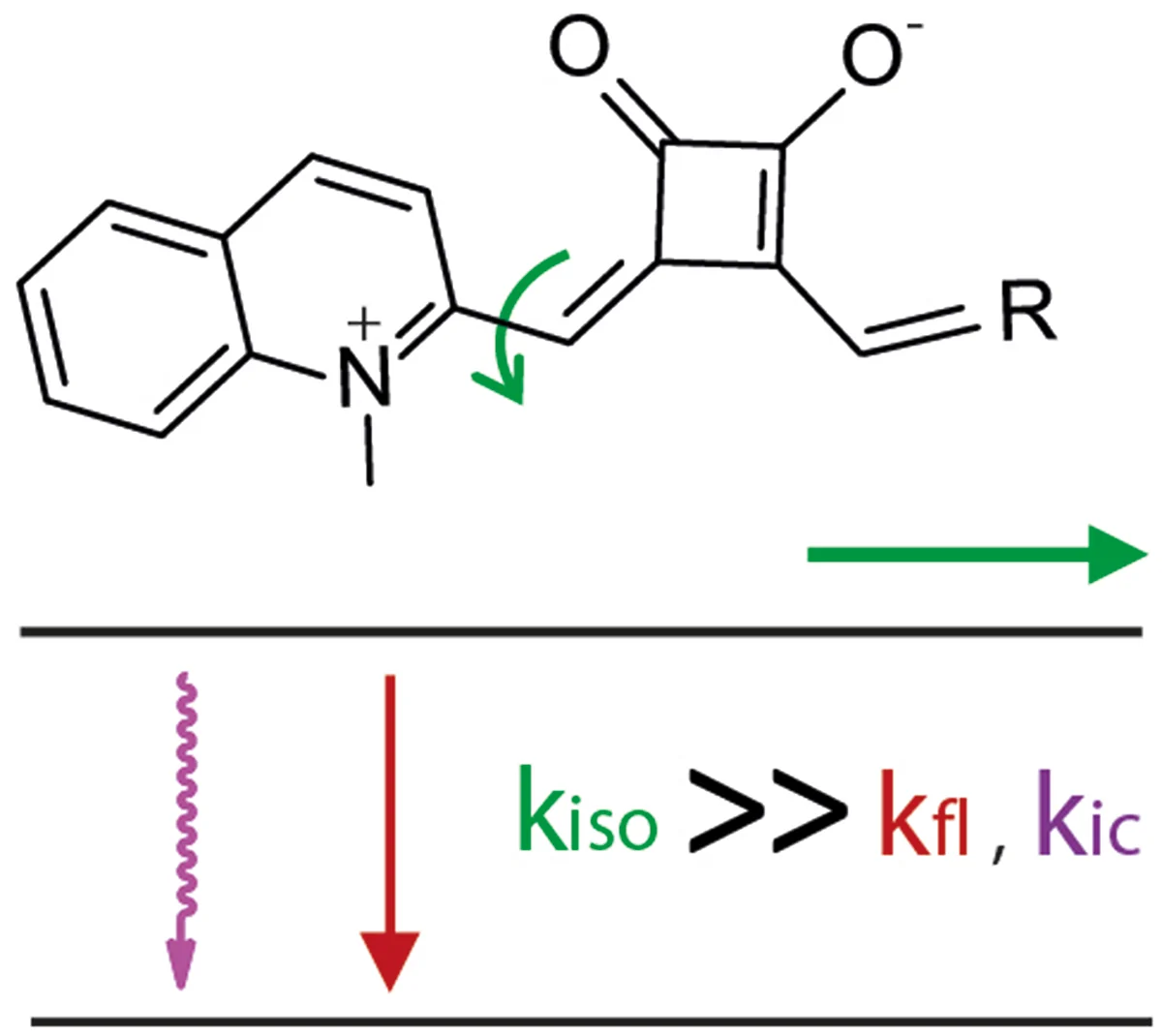

## Introduction

Squaraine (SQ) dyes are polymethine dyes that contain an electron-deficient central four-membered ring and two electron-donating groups in a donor–acceptor–donor (D–A–D) configuration with a resonance-stabilized π-conjugated structure. SQs comprise a wide variety of donor- and acceptor-substituted molecules [1–4]. In general, SQs are known for their straightforward and scalable synthesis, intense absorption and emission with a small Stokes shift, as well as good thermal- and photostability [5,6].

Methine-bridged SQs are synthesized by a condensation reaction of squaric acid or its derivatives with aromatic methylene base donor units [1–3,7,8]. 1,3-methine‑substituted SQs are cyanine-type chromophores [3], whereas 1,2-methine‑bridged SQs have a cross-conjugated π-electron system that spans from the donor to the carbonyl acceptor, and they can therefore be regarded as merocyanine-type chromophores [9–12]. 1,3-SQs display strong and narrow absorption in the far-red and near-infrared region, a property which has frequently been used for applications in solar cells and photodetectors [13–16]. In addition, various examples have used 1,3-SQs as sensors and molecular probes in fluorescence-based (bio-)imaging techniques [17–20]. Here, it is exploited that the SQ fluorescence sensitively depends on the local environment when the dye binds to a biomolecule. Similarly, the fluorescence quantum yield increases when the dye is encapsulated, which prevents aggregation and rigidifies the structure [21]. In contrast to the wide technological exploitation of 1,3-SQs, there are relatively few application cases and studies on the photophysical properties of 1,2-SQs, and these dyes are often considered merely as a side-product during synthesis [9,22].

The usefulness of SQs as fluorescence probes can often be related to the known photoisomerization reaction in polymethine dyes. Photoisomerization involves light-induced reversible changes between *cis* and *trans* geometric isomers, primarily occurring from the excited singlet state via rotation around bonds in the conjugated chain, creating a twisted intermediate that returns to the ground state isomer. The isomerization reaction acts as a non-radiative decay channel of the excited state and competes with fluorescence. The efficiency of photoisomerization is highly sensitive to the polarity and viscosity of the solvent as well as interactions with analyte molecules, which can retard or enhance the process.

Here, we synthesize a series of novel visibly absorbing and emitting 1,2-SQs and find that their photoluminescence quantum yield (PLQY) strongly depends on the specific donor moiety and the solvent properties. We compare PLQYs with values reported for other 1,2-SQs and include PLQYs from the related 1,3-SQ isomers. Because the electronic structure of 1,2-SQs (merocyanine-type) and 1,3-SQs (cyanine-type) is different, absolute PLQY values cannot be compared directly. However, we find for both dye classes that the presence of the quinoline-derived donor results in almost complete quenching of fluorescence. We note that this observation has (implicitly) also been made for a symmetric quinoline-heptamethine cyanine dye [23]. We discuss this finding in connection with the photoisomerization reaction occurring in these compounds. Efficient non-radiative deactivation is supported by quantum chemical calculations that reveal no energy barrier along the isomerization coordinate and a long bond around which the quinoline moiety rotates. Two simple design rules emerge finally from a simple three-state model to describe the electronic states of symmetric and non-symmetric D–A–D systems: A low PLQY is expected for asymmetric molecules when the difference between the two donors is large, whereas for symmetric dyes, radiationless decay is favored with strong donors that yield increasing flexibility of the excited state. The verifiability of these rules with the available PLQY data for SQs is discussed.

## Results

The SQ synthesis can be directed towards 1,2-SQs by using squaric acid esters and towards 1,3-SQs by using squaric acid [2,9]. For **1** (Scheme 1), the quinoline methylene base was reacted with squaric acid diethyl ester in a 1:1 ratio to afford the semisquarine. Subsequently, the semisquaraine was condensed with one equivalent of the benzothiazole methylene base to afford the unsymmetric 1,2-SQ **1** in 85% isolated yield. Several substituted quinoline and benzothiazole methylene bases were condensed in a similar manner to yield compounds **1a-1f**. Symmetric 1,2-SQ **2** was obtained in a one-step reaction by using 2 equivalents of the quinoline methylene base. In this case, the yield was only 20%, because the quinoline methylene base is less electron-rich than the corresponding benzothiazole methylene base. Compound **5** as well as the asymmetric target 1,2-SQ containing the quinoline- and indoline-derived heterocycle could not be synthesized with the chosen synthetic route [9]. 1,3-SQs were synthesized following literature procedures (Supporting Information, SI, Chapter 2).

**Scheme 1.**
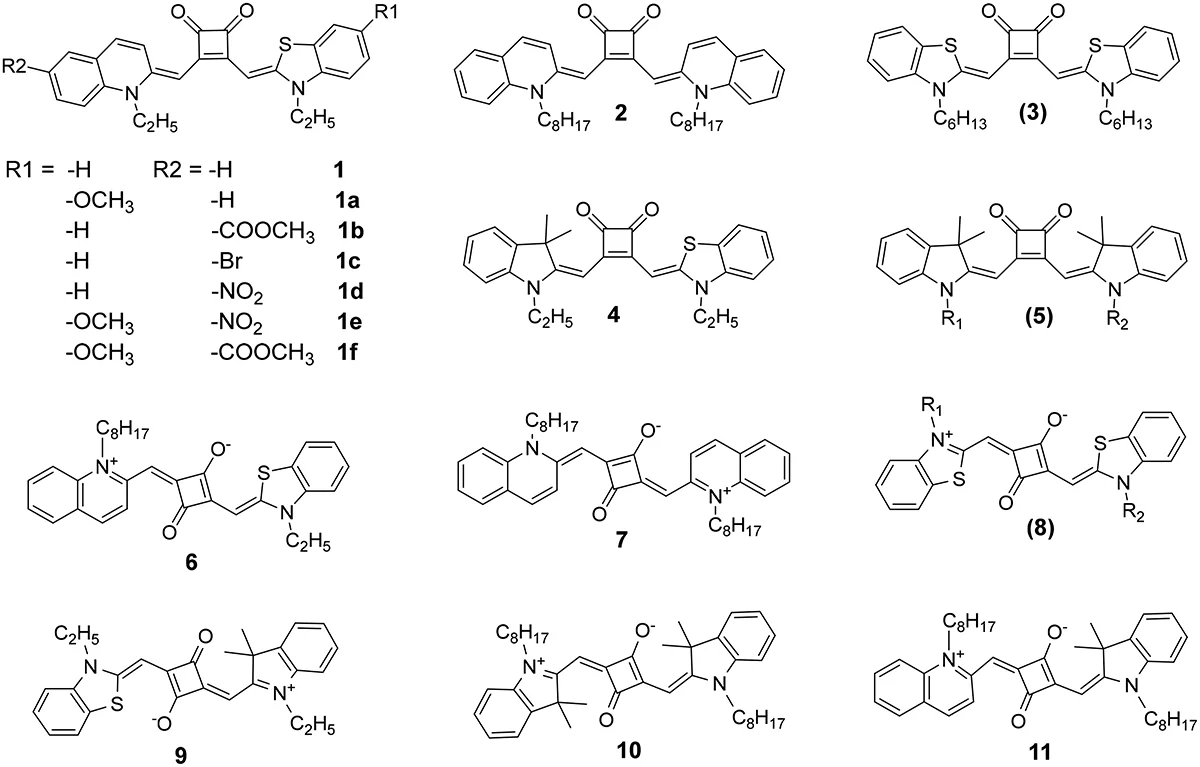
1,2-SQs (**1-5**) and 1,3-SQs (**6-11**) are incorporated in this work. Compounds in parenthesis (**3,5,8**) were not synthesized in this work. Compounds **1-1f** and **11** are reported for the first time.

The dye purity and the structural assignment were performed with NMR spectroscopy and mass spectrometry (SI, Chapter 2). Rotation around bonds along the conjugated chain results in dye stereoisomers [14]. Crystal structures for **3** [1,9] and calculations (Figure 1(a)) show that 1,2-SQs are most stable when adjacent double bonds and the central cyclobutene double bond are in the s-trans conformation, the nitrogen atoms of the donor groups face in the same direction (‘syn’), both away from the acceptor unit (‘out’). For 1,3-SQs, heterocycles are attached on opposite sides of the polymethine chain (‘trans’), and the nitrogen atoms are arranged in the ‘anti-out’ conformation (Scheme 1).

**Figure 1.**
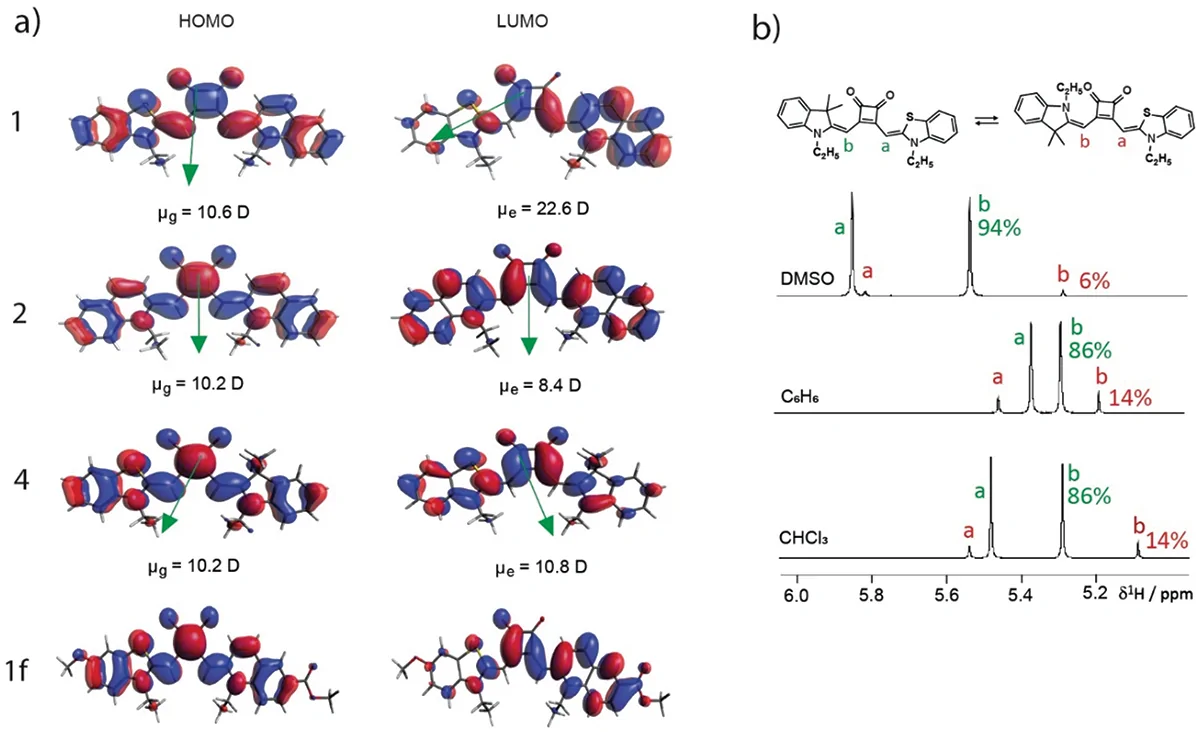
a) Frontier molecular orbitals of selected 1,2-SQs, calculated ground- and Franck–Condon excited-state dipole moments (μ [D, Debye]) are indicated for **1**, **2** and **4** by green arrows. b) ^1^H NMR spectra of **4** in different solvents.

For **4**, a reversible syn ↔ anti rotation at the indoline unit was observed from ^1^H NMR spectra, with the minor conformer varying between 6% and 14%, depending on the solvent (Figure 1(b)). No isomers were detected for other compounds from NMR spectra. When cooling a chloroform solution of **2** down to 248 K, rotation around the nitrogen-methylene bond slows down and the resonance of the -N-C**H**_2_-(CH_2_)_6_-CH_3_ protons splits into two separate signals, while the linewidths of other NMR signals of **2** remain narrow (SI, Figure S11). This indicates that at room temperature, the quinoline unit in 1,2-SQs does not rotate fast around bonds along the polyene chain, and this excludes the presence of a substantial amount of the molecular conformation where the nitrogen atom and the carbonyl group are nearby. This agrees with the calculated large energy difference of 24 kJ mol^−1^ between the favored s-trans-syn-out and the s-trans-anti conformation of **1**. On the other hand, the corresponding energy difference for **4** is only 8 kJ mole^−1^, and the Boltzmann population is 4%, in good agreement with the experiment.

Figure 2(a) shows absorption spectra of **1** in different solvents. Chapter 3 of the SI compiles optical spectra for all compounds synthesized in this work. The absorption spectrum of **1** in dichloromethane (DCM) is representative for the whole **1-1f** dye family. The spectra display a small positive solvatochromism (Table 1, Figure 1(a)), indicating stabilization of the more dipolar excited state (ES) by polar solvents (SI, Chapter 4) [24]. For selected derivatives **1a-1f**, methoxy donor groups were incorporated at position 6 of the benzothiazole moiety, combined with different acceptor groups at position 6 of the quinoline-derived unit. Calculations show that the HOMO is mainly located between the nitrogen atoms spanning the polyene scaffold, and that contributions on the terminal phenyl rings are minor (Figure 1(a)). This explains the small substitution effects on the absorption spectra for **1a-1f**, with the largest bathochromic shift (+23 nm) observed for **1e** (Table 1).

**Figure 2.**
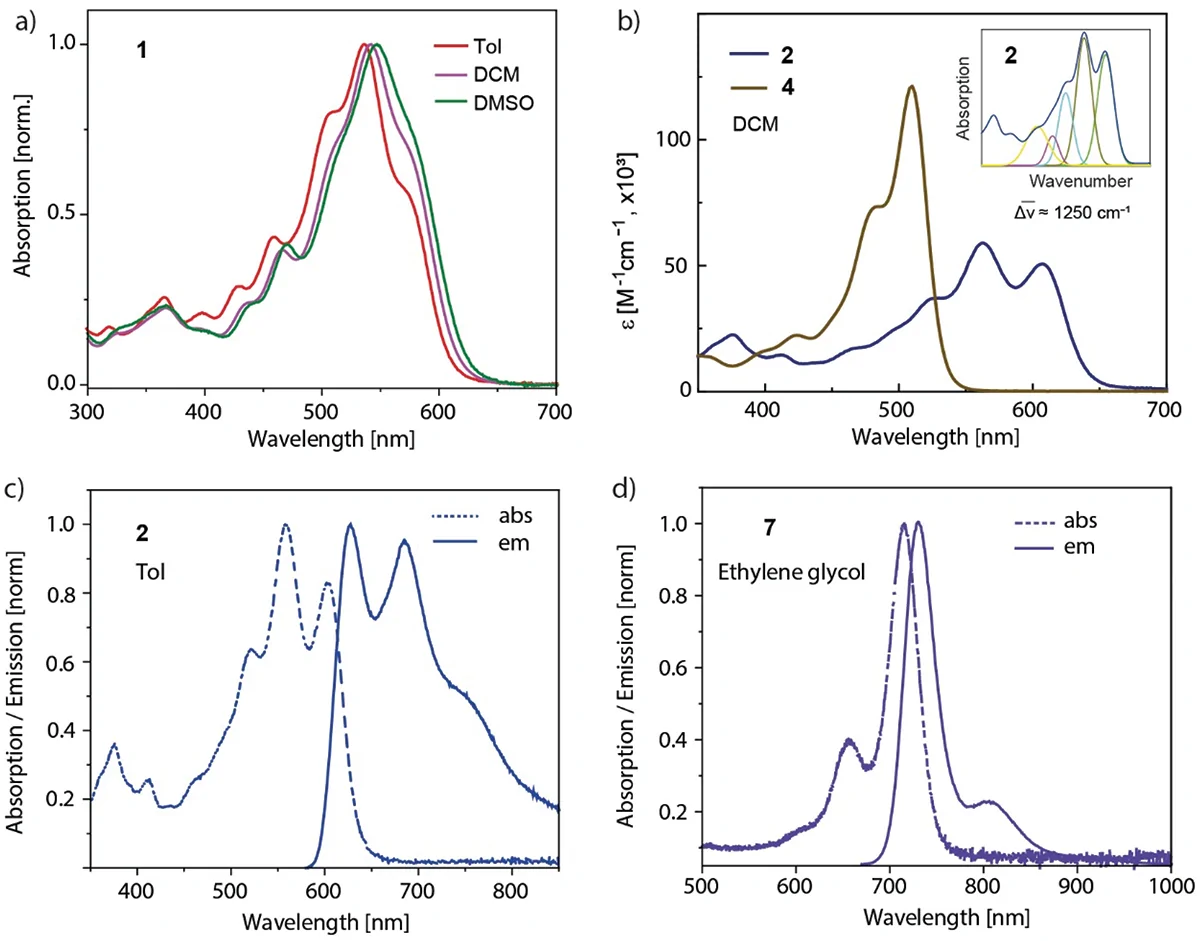
a) Absorption spectra of **1** in various solvents. b) Absorption spectra of **2** and **4** in DCM. c, d) Absorption and emission spectra of **2** and **7**.

**Table 1.** Absorption and fluorescence data for 1,2-SQs.

**Dye ^a)^**	**Solvent ^b)^**	**λ_abs_ [nm]** **at (ε_max_) ^c)^**	**Δν_1/2_** **[cm^−1^] ^d)^**	**λ_em,min_** **[nm]**	**Stokes shift** **[cm^−1^]**	**PLQY** **[%]**	**Remark**
**1**	Tol DCM DMSO EG ACN MeOH	536 ^f)^ 541 (60) 546 547 536 539	3150	605 616 ≈630	862 1127	2.6 x 10^−3^ 3.1 x 10^−3^ 0.01	PLQY rel ^h)^; λ_max,abs_ (0→0) 575 PLQY rel; λ_max,abs_ (0→0) 576 (fitted) PLQY abs ^g)^ λ_max,abs_ (0→0) slightly redshifts with increasing solvent polarity, the most ≈ +14 nm for DMSO and EG
**1a**	DCM	544 (65)		≈625		1.2 x 10^−3^	PLQY rel
**1b**	DCM	542 (62)		≈620		1.1 x 10^−3^	PLQY rel
**1c**	DCM	548 (70)		≈630		9.4 x 10^−4^	PLQY rel
**1d**	DCM	557 (52)				nq ^e)^	
**1e**	DCM	564 (53)				nq	
**1f**	DCM	548 (69)		≈630		8.6 x 10^−4^	PLQY rel; 546 (68) for the 5-methoxy derivative of **1f**
**2**	ACN DCM MeOH Tol Tol 284 K Tol 319 K DMSO Octanol Oct 283 K Oct 320 K EG EG 284 K EG 319 K	559 560 (63) 601 558 569 560 607	3440	634 633 629 625 644 632 634	838 731 741 584 839 706 702	0.02 0.02 0.02 0.03 0.033 0.024 0.04 0.08 0.12 0.05 0.12 0.18 0.07	λ_max,abs_ (0→0) 602; PLQY rel λ_max,abs_ (0→0) 605; PLQY rel PLQY rel λ_max,abs_ (0→0) 603; PLQY rel PLQY rel PLQY rel λ_max,abs_ (0→0) 611; PLQY rel λ_max,abs_ (0→0) 605; PLQY rel PLQY rel PLQY rel PLQY abs PLQY rel PLQY rel
**(3)**	Tol DCM EtOH ACN	515 522 (25) 525 519 (111)		529 539 543 542	514 604 631 818	2 4.4 4.4 0.7	[9] [9] [9] [10]; ε_max_ 41, PLQY 3.3% [9]
**4**	ACN DCM Tol DMSO Octanol EG	508 510 (121) 502 519 511 522	2030	531 518 538 530 546	853 615 680 702 842	1.3 0.5 4.0 6.2 13.0	PLQY abs; PLQY 2.9% [9] λ_abs_ 511; PLQY 2.9% [9] PLQY rel; λ_abs_ 504 [9]; ISC yield 6.5% (SI) PLQY rel; PLQY 22.8% [9] PLQY rel PLQY abs
**(5)**	EtOH	500 (79)					[28]

a) Data for compounds in parenthesis are taken from the literature; b) at ≈296 K if not specified, the polarity and viscosity of the solvents are compiled in the SI; c) [ε] = M^−1^cm^−1^, x10^3^; d) Δν_1/2_ is the FWHM of absorption, indicated for selected spectra; e) PLQY low, not quantified (nq); f) for compounds 1-1f the highest intensity is the 0 → 1 transition; g) absolute (PLQY abs) measurement of PLQY in integrating sphere; h) relative PLQY (PLQY rel), using as reference the same compound measured absolute at a different temperature or in a different solvent.

The absorption spectrum of **1** shows a broad Franck–Condon envelope with a well-resolved vibronic progression, a signature for 1,2-SQs that contain the quinoline moiety. During electronic excitation, the rather symmetric electron density in the HOMO is transferred away from the benzothiazole unit to the LUMO that is distributed on the quinoline and the central acceptor unit (Figure 1(a)). The electronic excitation couples to vibrational modes of the quinoline moiety, and for the symmetric dye **2**, the vibronic progression can be well approximated with an average vibronic spacing of around 1250 cm^−1^ (Figure 2(b), insert) [25]. For symmetrical 1,2-SQs, absorption maxima shift bathochromically in the order **5** (500 nm, ethanol (EtOH)) → **3** (525 nm, EtOH) → **2** (601 nm, methanol), which is ascribed to increasing π-conjugation for the donors indoline <benzothiazole <quinoline. In good approximation, the absorption of unsymmetrical 1,2-SQs lies in between the absorptions of the corresponding symmetrical dyes.

The absorption spectrum of 7 (Figure 2(d)) is typical for 1,3-SQs (SI, Chapter 3) [3,27]. Absorptions exhibit a small negative solvatochromism [6,14] (Table 2), whereas transient absorption spectra of the first ES of 1,3-SQs show a positive solvatochromism [26]. The main optical transition of 1,3-SQs exhibits a very high molar absorption coefficient ε and a narrow linewidth Δν_½_, with ε > 2.3 × 10^5^ M^−1^cm^−1^ and Δν_½_ ≈620 cm^−1^ for symmetric dyes **7** and **10**, and ε ≈1.6 × 10^5^ M^−1^cm^−1^ and Δν_½_ > 750 cm^−1^ for asymmetric **6**, **9** and **11**. Thus, the electronic asymmetry in 1,3-SQs is correlated with a broadening of the absorption band [6]. Absorption maxima for symmetrical 1,3-SQs (**10** (638 nm) → **8** (670 nm) → **7** (738 nm), all in DCM) agree with the sequence of donors observed for 1,2-SQs. Also, the absorption for unsymmetrical 1,3-SQs **6**, **9** and **11** lies halfway between the corresponding symmetrical dyes [6,8].

**Table 2.** Absorption and fluorescence data for 1,3-SQs.

**Dye ^a)^**	**Solvent ^b)^**	**λ_abs_ [nm]** **at (ε_max_) ^c)^**	**Δν_1/2_** **[cm^−1^] ^d)^**	**λ_em,min_** **[nm]**	**Stokes shift** **[cm^−1^]**	**PLQY** **[%]**	**Remark**
**6**	Tol DCM DMSO	725 709 (150) 714 (115)	1619 1580	743 732	334 443	nq ^e)^ 0.27	PLQY abs ^f)^ [29]
**7**	DCM MeOH Tol EG	738 (236) 708 752 715	630	751 723 765 730	235 293 226 287	0.13 0.19 0.24 1.50	PLQY abs in all solvents; λ_abs_ 738 [30]
**(8)**	Tol DCM MeOH ACN IP	678 670 649		690 663	257 325	53 9 21 13/31	[26]; PLQY 85% [31]; 193 cm^−1^ [32] [6]; 264 cm^−1^ [32] [6]; PLQY [26] [31] [6]/[26]
**9**	DCM	651 (177)	765	662	255	33	PLQY abs; PLQY 10% IP, λ_abs_ 635 MeOH [6]
**10**	Tol Tol 285 K Tol 320 K DCM EG EG 287 K EG 320 K EtOH ACN	642 638 (290) 638 631 634	615	652 652 647 641 643	239 337 218 247 221	50 ± 2 58.1 33.7 21±0.4 39 49 22 17 12	PLQY abs; PLQY 38% [33], 58% [34] PLQY rel ^g)^ PLQY rel PLQY abs; λ_abs_ 636 (357) [35]; PLQY 27% [35], 13% [33], 27% [34] PLQY abs PLQY rel PLQY rel PLQY abs; PLQY 8% [33], 15% [34] PLQY abs; PLQY 6% [34], 5% [33]
**11**	DCM	690 (176)	1580	715	507	0.23	PLQY abs

a) data for compounds in parenthesis are taken from literature; b) at ≈296 K, if not specified; c) [ε] = M^−1^cm^−1^, x10^3^; d) Δν_1/2_ is the FWHM of absorption, indicated for selected spectra; e) PLQY low, not quantified (nq); f) absolute (PLQY abs) measurement of PLQY in integrating sphere; g) relative PLQY (PLQY rel), using as reference the same compound measured absolute at a different temperature or in a different solvent.

Emission spectra of 1,2-SQs are in good approximation mirror images of the absorption (Figure 2(c)) and Stokes shifts are small and rather insensitive to the solvent (Table 1 and SI, Chapter 3). In DCM, the reported [9] PLQY is highest for **3** and amounts to ≈4%. For **4**, the PLQY increases with solvent viscosity. Thus, the PLQY is 0.5% in toluene and increases to 13% in ethylene glycol (EG). On the other hand, there is no correlation between the PLQY of **4** and solvent polarity. The same trends are observed for **2**, and the PLQY in acetonitrile (ACN, 0.02%) increases to 0.12% in EG. Cooling down (EG, 284 K) increases the PLQY to 0.18%, confirming that the emission increases with viscosity. Note that PLQY values of **1** and **2** are indeed very small.

Also, the emission spectra of 1,3-SQs are in good approximation mirror images of the absorption (Figure 2(d) and SI, Chapter 3), and Stokes shifts (≈200–500 cm^−1^) are small (Table 2). PLQYs for compounds **8-10** have been reported before. Experimental values from different laboratories scatter notably, but the general trend is that the PLQY in non-polar solvents is high and drops with increasing solvent polarity. In addition, the PLQY increases with solvent viscosity. Thus, the PLQY for **10** in EtOH is 17%, but increases to 39% in EG. EG has the same polarity as EtOH but a much higher viscosity. When lowering the temperature in EG to 287 K, thus increasing the solvent viscosity further, the PLQY arrives at 49%. As observed for 1,2-SQs, the PLQY drops strongly for 1,3-SQs **6**, **7** and **11** that contain the quinoline unit. PLQYs are below 0.5% and the emission yield increases to 1.5% in EG for **7**, again indicating a positive viscosity effect.

## Discussion

Small experimental, solvent-independent Stokes shifts and mirror images of absorption and emission spectra indicate minor electronic reorganization effects and small geometric changes between the ground state (GS) and ES. Small reorganization reduces the opportunity for non-radiative relaxation and increases the likelihood of radiative relaxation via fluorescence [20,31,36–38]. In contrast, selected SQs show a pronounced positive PLQY dependence on solvent viscosity (**2**, **4**, **7**), a negative relation with solvent polarity (**8**, **10**), and the presence of the quinoline donor unit in both 1,2-SQs and 1,3-SQs quenches the emission by around two orders of magnitude (Tables 1 and 2). The PLQY is defined as the ratio between the radiative rate (k_f_) and the sum of radiative and non-radiative excited state relaxation rates (∑k_nr_) by which the ES (*S*_*1*_) deactivates, PLQY = k_f_/(k_f_ + ∑k_nr_). As possible non-radiative loss channels, we consider *S*_*1*_ → ^*3*^*S*_*1*_ intersystem crossing (k_ISC_), *S*_*1*_ → *S*_*0*_ internal conversion through vibrational modes (k_ic_), and the photoisomerization reaction from *S*_*1*_ that involves twisting motion of a lateral donor group in the polymethine bridge (k_iso_). We argue that the observed PLQY trends are dictated by the reactive ES decay via bond twisting and rotation to lower energetic molecular configurations, i.e. k_iso_ > k_ISC_, k_ic_.

There is ample experimental evidence that triplet formation for 1,3-SQs containing oxygen atoms in the squaric unit is close to zero [26,34–36,39]. Calculations in reference [22] suggest that ISC in 1,2-SQs containing the benzothiazole unit occurs. Furthermore, the energy of the 1,2-SQ triplet state is sufficiently high (>1 eV) so that energy transfer to oxygen is possible. This allows approximate quantification of the ISC yield via the reaction sequence ^*3*^*SQ*_*1*_ + ^*3*^*O*_*2,o*_ → *SQ*_*o*_ + ^*1*^*O*_*2,1*_; ^*1*^*O*_*2,1*_ → photoluminescence at 1270 nm [40–42]. Using compound **4**, we measured substantial singlet oxygen emission in air-saturated toluene solution, and quantification in comparison with the reference compound benzophenone resulted in an ISC yield of ≈6.5% for **4** (SI, Chapter 5). No emission was observed when testing compound **1**, which contains both the benzothiazole and quinoline donor. We interpret this that in this case, ISC cannot compete with the much faster isomerization reaction, i.e. k_iso_ >>k_ISC_. Thus, ISC occurs in 1,2-SQs and contributes to some degree to non-emissive deactivation, but it does not explain the strong PLQY falloff observed for **1** and **2**.

Similarly, internal conversion is an important non-radiative ES decay process but probably does not dictate the observed PLQY trends. Insertion of the quinoline unit in the dye results in a bathochromic shift (≈50–80 nm) of the emission. When approximating the GS and ES energy surfaces as harmonic potentials, k_f_ decreases as the third power with decreasing emission energy, whereas k_ic_ increases faster than exponential (the energy gap law [43]). Therefore, the PLQY of quinoline-containing compounds is expected to be lowered. However, for the cyanine dye family, it has been shown that the unavoidable steep PLQY falloff due to the energy gap law starts only around 900 nm [38]. This range is remote from the emissions of 1,2-SQs **1** and **2** (below 650 nm) and 1,3-SQ **6**, **7** and **11** that emit still in the far-red, below ≈750 nm. Also note that for **8** and **10**, the PLQY decreases with increasing emission energy, which contrasts with the energy gap law [44].

We discuss photoisomerization (k_iso_) as the dominant non-radiative decay channel for our dye series [33,34,45,46]. When the molecule rotates out of planarity, the energy of the GS continuously increases along the isomerization coordinate (ρ), while the energy of the ES with increasing torsional motion enters a local energy minimum as ρ approaches 90°. In this geometry, the energy gap between the GS and ES is small, providing an effective deactivation path to the dye in the GS or to a photoisomer. While this overall picture is accepted for polymethine dyes, energetic details of the topography of the potential energy surfaces (PES) and the extent of charge transfer (CT) in the twisted ES are subtle and molecule-specific [47–50]. For example, photoisomerization can be an activated process or can proceed barrierless [51]. Similarly, the decay process can proceed ultrafast via a conical intersection (CI), or the GS and ES in the twisted geometry may rest in a region of considerable energy gap, in which case non-radiative decay competes with ISC or fluorescence [52–55].

The branching ratio between fluorescence and isomerization is influenced by the height and dipole moment of the barrier on the path from the Franck–Condon ES to the twisted geometry. For a merocyanine dye (model compounds for 1,2-SQs), it was found from theoretical investigation that the ^*1*^*S*_*1*_ region had a larger dipole moment than the GS, the transition state and the CI. Therefore, the energy of the ^*1*^*S*_*1*_ state was lowered most by polar solvents, and access over the barrier to the CI was restricted with increasing solvent polarity. This explained the strong PLQY increase observed with increasing solvent polarity [24]. In another example, the variation of the CI energy for a series of dicyano-distyrylbenzenes was less than the Franck–Condon energy between the different compounds. Therefore, the energy barrier from the Franck–Condon region to the CI increased with increasing absorption wavelength. Consequently, access to the relaxation channel was hindered and k_iso_ decreased for compounds with bathochromic absorption [44]. This has been termed as ‘inverted energy gap law’ behavior; in our case, solvent-dependent PLQYs for **8** and **10** match this trend (see above) [24,44,50]. The photoisomerization of **10** (with various N-alkyl and aromatic substituents) has been studied before [33,34,36,46]. Results suggest that photoisomerization in the ES involves a barrier with an increased dipole moment and that rotation occurs at the carbon bond next to the indoline unit. Non-radiative deactivation from the twisted geometry mostly regenerates the trans-anti-out conformation in the GS directly, but transient GS photoisomers that should correspond to the trans-syn conformation have been detected, which convert to the stable stereoisomer on the μs–ms time scale [34,36]. Our analysis does not permit any statement on the relative population of GS photoisomers when the ES deactivates.

The presence of a polar barrier along the reaction coordinate for **10** explains the experimental PLQY increase with decreasing temperature and decreasing solvent polarity (Table 2). For **10** and **8**, there is also no clear PLQY difference between polar protic and polar non-protic solvents; thus, specific hydrogen bonding interactions between the solvent and the oxygen atoms on the cyclobutene ring that influence the PLQY are of minor importance for these dyes. However, in parallel to the accepted photoisomerization decay channel, a deactivation route has been proposed that involves stretching of the carbonyl bond of the squaric unit [46].

Qualitative insight into the height and polarity of the isomerization barrier is obtained from temperature-dependent PLQY measurements [33,34]. We assume there is an energy barrier (E_b_) due to solvent viscosity, which slows down large‑amplitude motions during photoisomerization, and that E_b_ is proportional to the activation energy (E_ƞ_) of the solvent viscosity [56]. The barrier E_b_ adds to the intrinsic photoisomerization barrier E_i_. The height of E_i_ depends on the polarity of the transition state, and E_i_ for a polar barrier is lowered in a polar solvent. For **2** and **10**, we measured fluorescence in toluene and EG in the temperature range 285 K − 320 K (SI, Chapter 6). With the calculated radiative rates k_f_ [25], we evaluated k_iso_ = k_f_(1/PLQY −1) and obtained apparent activation energies E_a_ ≈ E_ƞ_ + E_i_ from the slopes of the Arrhenius plot.

For **10**, E_a_(Tol) ≈ 21 kJ mol^−1^ was much larger than E_ƞ_(Tol) ≈ 9 kJ mol^−1^, which indicates that photoisomerization involves an intrinsic activation barrier. On the other hand, the apparent and solvent viscosity activation energies for **10** were similar in EG, E_a_(EG) ≈ E_ƞ_(EG) = 29 kJ mol^−1^, indicating a small E_i_ in this solvent. This agrees with the large dipole moment of the transition state for **10** during isomerization, which is stabilized and lowered in the more polar solvent EG.

The behavior of **2** was different, and the apparent activation energies E_a_(Tol) = 7 kJ mol^−1^, E_a_(EG) = 20 kJ mol^−1^ were smaller than E_ƞ_ for both solvents. The calculated dipole moments for the GS and Franck–Condon ES of **2** (Figure 1(a)) are smaller than the dipole moment during twisting (see below). In a simplified description, this insensitivity to the solvent polarity and the almost complete fluorescence quenching of **2** observed in any solvent then indicates that E_i_ is very small. In this case, isomerization is a fast barrierless downhill process and is opposed by the viscous drag of the surrounding solvent [51].

We computed PES profiles along the isomerization coordinate with time-dependent density functional theory (TD-DFT) calculations (SI, Chapter 1). For **10**, the results indicate that bond rotation proceeds via a barrier with a height of ≈ 400 meV in the gas phase (Figure 3(a)). In addition, the calculated dipole moment during twisting increases to ≈11 D, whereas the dipole moment of **10** in the GS and Franck–Condon ES is zero (C_2 h_ symmetry). The dipole moment of the transition state is stabilized in a polar environment, which explains that the calculated barrier height decreases with increasing solvent polarity (Figure 3(a)). These calculated features agree with the experimental evidence, which confirms the qualitative accuracy of the selected level of theory for our purpose; details of the twisted intermolecular CT region where the ES relaxes to the GS cannot appropriately be addressed with TD-DFT calculations [24,44].

**Figure 3.**
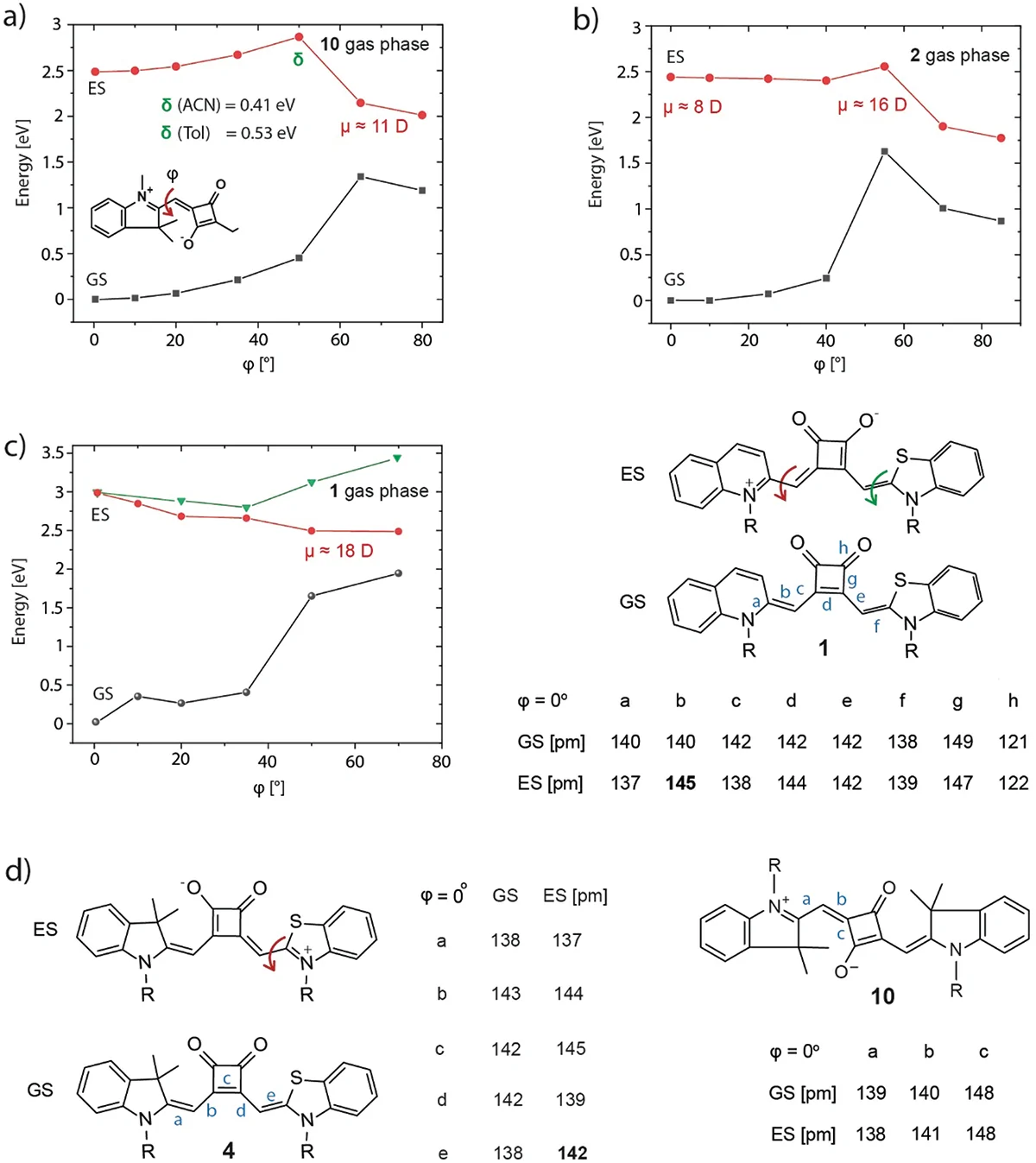
Calculated energy profiles along the isomerization coordinate (ρ) for (a) **10**, (b) **2** and (c) **1**. Selected calculated dipole moments μ are indicated, and in (a), δ indicates the barrier height along the isomerization coordinate. c, d) Calculated bond lengths in the ground state (GS) and relaxed excited state (ES).

For **2** (Figure 3(b)), the calculated barrier height is much smaller than for **10**, and the barrier is minor when twisting the quinoline unit in **1** (Figure 3(c)). On the other hand, rotation of the benzothiazole unit in **1** is energetically disfavored, and the calculated ES energy continuously increases with twisting angle. Again, these results from approximate calculations confirm experimental findings and indicate that rotation around the quinoline donor unit in the ES of SQs is (almost) barrierless and therefore fast, which quenches the PLQY.

We attempt to give an intuitive explanation for k_iso_, which decreases in the order quinoline > benzothiazole >indoline. Therefore, we consider the calculated bond lengths in the GS and ES of **1**, **4** and **10** (Figure 3(c,d)). For **1**, the bond lengths indicate that the GS is dominated by the neutral resonance structure, as drawn in Figure 3(c). Upon photoexcitation, electron density is transferred from the benzothiazole to the quinoline moiety (Figure 1(a)), and the ES is dominated by the zwitterionic resonance structure between the quinolinium cation and the alkoxide anion over the polyene chain. Bond lengths remain constant in the benzothiazole donor part of **1** upon excitation, but we mention the elongated bond length (145 pm) in the ES next to the quinolinium moiety, via which isomerization occurs [24,50].

In **4**, electron density is transferred from the indoline to the benzothiazole part upon excitation (Figure 1(a)), and the ES in this case is dominated by a zwitterionic resonance structure between the benzothiazolium cation and the alkoxide anion. The bond next to the benzothiazole moiety is also extended in the ES but remains shorter (142 pm, Figure 3(d)) than the isomerization bond in **1**.

The significantly elongated bond adjacent to the quinoline moiety in the ES of **1** allows facile (and therefore rapid) torsional motion, while in **4**, the isomerization bond exhibits more double-bond character and rotation becomes energetically costly, impeding access to the twisted ES. This picture is compatible with a simple three-state model (Essential States Model, ESM) initially proposed by Terenziani *et al*. [57] to describe the electronic states of symmetric D–A–D systems, and later extended in reference [32] to non-symmetric D_1_–A–D_2_ systems. We qualitatively discuss here both cases, neglecting some numerical details of such models, as they are not necessary to interpret the experimental and computational data presented above.

The electronic states of the system can be described in terms of three diabatic configurations (Figure 4(a)): a neutral one |*N* > = |*D*_*1*_*AD*_*2*_ >, and two zwitterionic ones, |*Z*_*1*_ > = |*D*_*1*_^*+*^*A*^−^*D*_*2*_ > and |*Z*_*2*_ > = |*D*_*1*_*A*^−^*D*_*2*_^*+*^ > . Off-diagonal matrix elements *t*_*1*_ and *t*_*2*_ describe the efficiency of electron transfer from each donor to the acceptor (Figure 4(b)). In a symmetric dye, the *Z*_*1*_ and *Z*_*2*_ configurations are degenerate. Diagonalization of the ESM Hamiltonian shows that the first ES of the system is described by a symmetric combination of these two zwitterionic configurations. This leads to negligible dipole change upon excitation. This picture is supported by TD-DFT/M06-2X/def2-SVP calculations: for both dyes, analysis of the *S*_*0*_ *→ S*_*1*_ transition provides a symmetric distribution of both the HOMO and the LUMO (Figure 1), highlighting no charge centroid shift upon transition (*Δr* ~0.05 Å, where *Δr* is the hole-electron distance upon excitation [58]).

**Figure 4.**
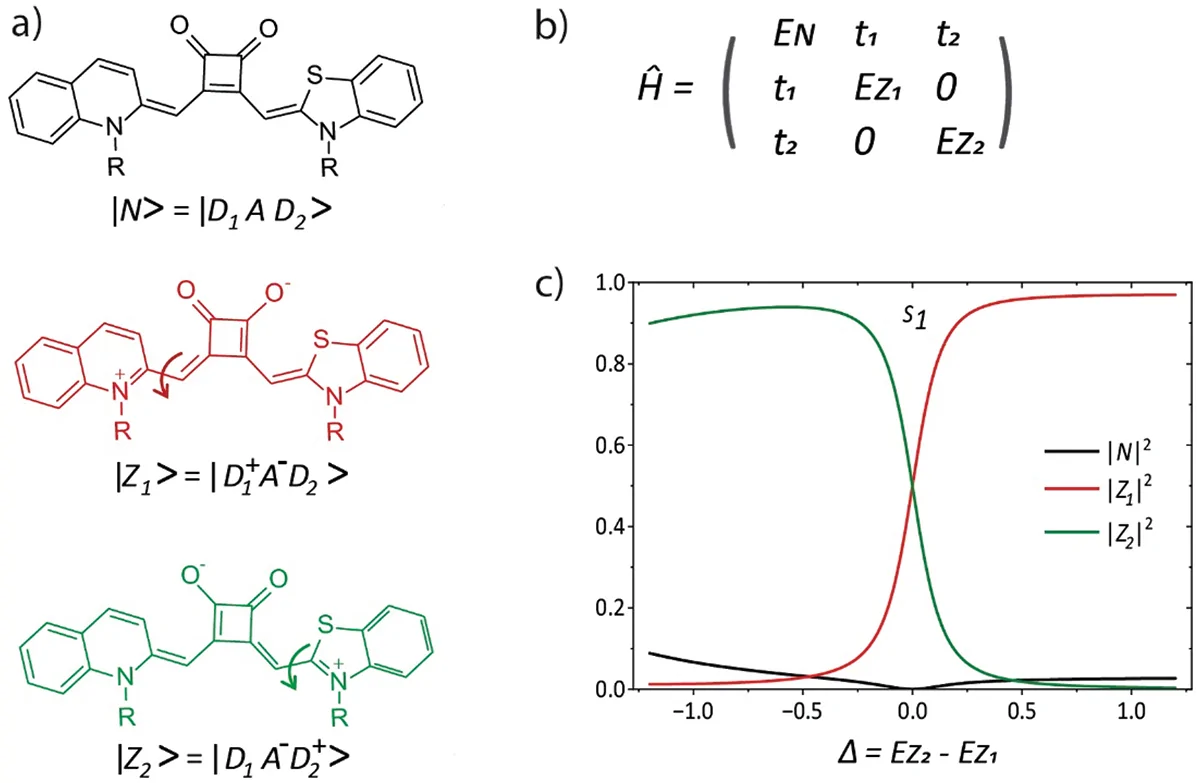
Electronic Essential States Model describing a D_1_–A–D_2_ system, here compound **1**. a) Diabatic configurations. b) Hamiltonian. c) Weights of the diabatic configurations in the first excited (eigen) state of the Hamiltonian.

The picture changes substantially when the two donor moieties are chemically non-equivalent. In this case, the *Z*_*1*_ and *Z*_*2*_ configurations are no longer degenerate and cannot mix as efficiently, so the adiabatic states resulting from diagonalization retain a character closer to the individual diabatic configurations. This confers to the first ES a CT character dominated by the strongest donor, a different dipole with respect to the GS and, therefore, (a slightly) stronger solvatochromism (Tables 1 and 2). In Figure 4(c), we diagonalise the ESM Hamiltonian keeping *E*_*N*_ (0 eV) and *E*_*Z1*_ (2.2 eV) fixed, scanning *E*_*Z2*_ (from 1.0 to 3.4 eV). We show the weights of each diabatic configuration as a function of the energy difference Δ = *E*_*Z2*_ - *E*_*Z1*_: the *S*_*1*_ electronic character is dominated by the lowest energy *Z* configuration (when Δ < 0, |*Z*_*2*_ > is at lower energy, when Δ > 0, |*Z*_*1*_ > is at lower energy). This qualitative interpretation is confirmed for molecule **1** by TD-DFT/M06-2X/def2-SVP calculations: analysis of the *S*_*0*_ *→ S*_*1*_ transition reveals a markedly non-symmetric LUMO with stronger involvement of the quinoline moiety, consistent with the dominant |*Z*_*1*_ > character predicted by the ESM (Figure 1). The CT nature of *S*_*1*_ is demonstrated by a significant charge centroid shift upon transition, typical of CT states (*Δr* ~1.64 Å). The identification of the quinoline moiety as a stronger donor with respect to benzothiazole is supported by experimental spectroscopic evidence on symmetric dyes **2** and **3**: the *S*_*0*_ *→ S*_*1*_ transition in the absorption spectrum of **2** is more red shifted in comparison to that of **3** (Table 1). Since such transition is dominated by a HOMO → LUMO excitation and the acceptor moiety is the same, the red shift observed in **2** with respect to **3** can be attributed to a destabilization of the HOMO, belonging to the donor (quinoline in **2** and benzothiazole in **3**), demonstrating that the quinoline-derived donor is stronger than the benzothiazole-derived one because of its higher HOMO energy (i.e. smaller ionization potential).

The non-symmetry due to different donor moieties has a profound impact on the photophysics of the system: the double bond connecting the strongest donor to the acceptor is strongly weakened in *S*_*1*_ (it is a single bond in the lowest energy *Z* configuration), and a rotation about such bond is possible, leading to a twisted CT geometry. This is demonstrated by our PES scans on molecule **1** reported in Figure 3, where the rotation of the quinoline moiety is barrierless on *S*_*1*_, while the benzothiazole has a barrier on the same PES: the *S*_*1*_ electronic state is dominated by the *Z*_*1*_ configuration, while *Z*_*2*_ hardly contributes. The twist about such bond, although favorable to stabilize the *Z*_*1*_ configuration, also leads to the proximity of a CI, promoting radiationless decay [59–61].

In light of this description, we can qualitatively conclude that the larger the difference between the two donors, the stronger the CT character of *S*_*1*_, and therefore the lower the PLQY. We denote this as ‘design principle 1’ (*DP1*). On the contrary, when the dye is symmetric (Δ = 0) and *S*_*1*_ is described by a symmetric mixture of the two zwitterionic configurations, the CT character is negligible. In this case, the double bonds connecting donors to acceptors partially retain their double-bond character in *S*_*1*_, as illustrated by the calculated bond lengths of molecule **10** (Figure 3(d)). As a result, access to the twisted geometry, where nonadiabatic couplings are strong, may be prevented by a barrier on the *S*_*1*_ PES (Figure 3(a)); the magnitude of such a barrier modulates the observed PLQY. As an example, we discuss in more detail molecules **2** and **3**, both symmetric D–A–D dyes, the latter showing PLQY ≈100 times larger than the former (Table 1). Experimental data show an increase of the PLQY of **2** in viscous solvents, hinting at a dihedral twisting motion driving radiationless decay. *S*_*1*_ PES relaxed scans carried out at TD-DFT/M06-2X/def2/SVP for molecules **2** and **3** show that in the former case, the barrier for the dihedral twist is ≈130 meV, while in the latter, it exceeds 300 meV (SI, Figure S1). Given that thermal energy at room temperature is *kT* ≈25 meV, the barrier in molecule **2** is low enough to be substantially crossed through thermal fluctuations, whereas the much larger barrier in molecule **3** effectively suppresses access to the twisted geometry. This qualitatively accounts for the observed difference in PLQY and hints to a general *DP2* for symmetric D–A–D dyes: the donor strength can be selected to tune the PLQY, with weak donors providing fluorescent dyes and strong donors yielding increased flexibility in *S*_*1*_, which are more easily subject to radiationless decay.

Given the present data availability for PLQY values of visible SQs, the conclusive testing of the two derived design principles (*DP1,2*) is difficult. From Tables 1 and 2, we extract for *DP1* that the PLQY of **1 < 4**, and of **6** ≈ **11** < **9**; for *DP2*, the PLQY of **2** < **3**, and of **7** < **8** ≈ **10**. Here, a clear assessment is hindered because compounds **1**, **2**, **6**, **7**, and **11** contain the very strong quinoline-derived donor which quenches the fluorescence in any case.

Published PLQY values are available for symmetric SQs where the indole-derived donors were replaced by benzo[e]indole-derived donors (SI, Chapter 7). This increases the donor strength and results in a bathochromic absorption shift of ≈+30 nm. The same bathochromic shift occurs when replacing the benzothiazole donors with naphthothiazole donors (SI, Chapter 7). However, this did not result in a clear decrease of the reported PLQYs. The main reason for this inconclusive trend seems to be the large PLQY variations for the same dye when measured in different laboratories, and the data spreading is probably larger than what is expected from *DP2*. Therefore, comparison along these lines should be performed via PLQY measurements carried out in the same laboratory. It should finally be mentioned that next to the proposed design principles other factors affect the PLQY of SQs. For example, twisting of the benzothiazole moiety is hindered by a positive dispersion interaction between the central squaric acid oxygen and the sulfur atom of the benzothiazole, estimated to ≈15 meV for **1** (SI, Chapter 1). Also, the bulkiness and steric stress of the rotating group need to be considered when comparing different donor groups. Here, a comparison of the PLQY of **10** with related (but so far not reported) dyes that contain none or more bulky substituents at the indoline 3-position would be interesting.

## Conclusions

We synthesized a series of new 1,2- and 1,3-methine-bridged SQs comprising different donor heterocycles and compiled a comprehensive set of SQ absorption and emission data, in various solvents and as a function of temperature. Absorptions for symmetrical compounds red-shift with increasing π-conjugation of the donors in the order quinoline >benzothiazole >indoline, and unsymmetrical dyes absorb halfway between the symmetrical dyes, confirming the expected trend. The most interesting observation is that fluorescence in any compound that contains the strong quinoline moiety is quenched by more than two orders of magnitude. We ascribe this to very efficient non-radiative decay via photoisomerization, which competes with emission of light from the excited state. Results from the frequently used 1,3-SQ dye family show that fluorescence quantum yields in the visible vary from >50% down to <0.2%; this implies that the remark ‘SQ dyes exhibit strong fluorescence in solution’ – quite frequently found in introductory chapters of scientific papers – should not be used in the general case. We finally mention that our discussion is only pertinent to SQs that emit in the visible and far-red. For SQs that emit beyond ≈900 nm, the low fluorescence quantum yield is usually explained by the small band gap and the concomitant, unavoidable increased coupling of the excited state to higher vibrational levels of the ground state, which deactivates the excited state via internal conversion with rate k_ic_ (the energy gap law). However, due to the very fast isomerization rate (k_iso_) in quinoline-SQs, it is conceivable that even in the near-infrared region isomerization contributes to excited state deactivation (i.e. k_iso_ ≈k_ic_). An indication for this is received from a recent fluorescent study on heptamethine cyanine dyes [23]. Here, the dye with terminal quinoline moieties showed strongly suppressed fluorescence, despite the emission being blue-shifted by more than 150 nm compared to dyes with other donor heterocycles.

## Data Availability

The authors confirm that the data supporting the findings of this study are available within this article and its supplementary information.
